# WITHDRAWN: Clinical implication of the patient's disease awareness and adherence to medications in patients undergoing atrial fibrillation ablation

**DOI:** 10.1002/joa3.70135

**Published:** 2025-07-21

## Abstract

**WITHDRAWN**: SawadaM. MD, OtsukaN. MD, PhD, NagashimaK. MD, PhD, WatanabeR. MD, PhD, WakamatsuY. MD, PhD, HayashidaS. MD, PhD, HirataS. MD, HirataM. MD, KurokawaS. MD, PhD, OkumuraY. MD, PhD, Clinical implication of the patient's disease awareness and adherence to medications in patients undergoing atrial fibrillation ablation, Journal of Arrhythmia 40, no. 1 (2023): 57‐66, 10.1002/joa3.12965 38333379 PMC10848582

The above article, first published online on 6 December 2023, on Wiley Online Library (onlinelibrary.wiley.com), has been withdrawn by agreement between the authors, the Editors in Chief Kazuo Matsumoto and Young‐Hoon Kim, the Japanese Heart Rhythm Society, and John Wiley and Sons Australia Ltd. The withdrawal has been made because the authors had not obtained permission to use the MMAS‐8 scale reported in the article. The authors apologize for this error.

